# Río Negro virus (Venezuelan equine encephalitis virus complex) shows pathogenic potential in a permissive murine model

**DOI:** 10.3389/fcimb.2026.1845208

**Published:** 2026-06-11

**Authors:** Luciana A. Fassola, Yamila Gazzoni, Glenda D. Martin Molinero, Soledad De Olmos, María F. Triquell, Alicia Degano, Marianela C. Serradell, María E. Rivarola, Sergio R. Oms, Marta S. Contigiani, Adriana Gruppi, Guillermo Albrieu-Llinás

**Affiliations:** 1 Centro de Investigación y Desarrollo en Inmunología y Enfermedades Infecciosas (CIDIE), Consejo Nacional de Investigaciones Científicas y Técnicas/Universidad Católica de Córdoba, Córdoba, Argentina; 2 Facultad de Ciencias Agropecuarias, Universidad Católica de Córdoba, Córdoba, Argentina; 3 Departamento de Bioquímica Clínica, Facultad de Ciencias Químicas, Universidad Nacional de Córdoba, Córdoba, Argentina; 4 Centro de Investigaciones en Bioquímica Clínica e Inmunología (CIBICI), Consejo Nacional de Investigaciones Científicas y Técnicas, Córdoba, Argentina; 5 Departamento de Química Biológica “Ranwel Caputto”, Facultad de Ciencias Químicas, Universidad Nacional de Córdoba, Córdoba, Argentina; 6 Centro de Investigaciones en Química Biológica de Córdoba (CIQUIBIC), Consejo Nacional de Investigaciones Científicas y Técnicas, Córdoba, Argentina; 7 Instituto de Investigación Médica Mercedes y Martín Ferreyra (INIMEC), Consejo Nacional de Investigaciones Científicas y Técnicas, Universidad Nacional de Córdoba, Córdoba, Argentina; 8 Departamento de Biología Celular, Histología y Embriología, Facultad de Ciencias Médicas, Universidad Nacional de Córdoba, Córdoba, Argentina; 9 Departamento de Fisiología, Facultad de Ciencias Exactas, Físicas y Naturales, Universidad Nacional de Córdoba, Córdoba, Argentina; 10 Instituto de Virología “Dr. JM Vanella”, Facultad de Ciencias Médicas, Universidad Nacional de Córdoba, Córdoba, Argentina; 11 Facultad de Ciencias de la Salud, Universidad Católica de Córdoba, Córdoba, Argentina

**Keywords:** Alphavirus, neuroinvasion, pathogenesis, Río Negro virus, Venezuelan equine encephalitis virus complex

## Abstract

Río Negro virus (RNV), an enzootic member of the Venezuelan equine encephalitis virus (VEEV) complex, has historically been regarded as avirulent based on studies in adult immunocompetent animals. However, its pathogenic potential under biologically relevant conditions has not been systematically examined. Here, we investigated RNV infection in infant C57BL/6 mice as a permissive developmental model. Infection resulted in a transient viremic phase followed by systemic dissemination, disruption of splenic immune architecture, and pronounced neuroinflammatory and neurodegenerative alterations in the central nervous system. Viral replication in brain tissue coincided with astrocyte activation and elevated pro-inflammatory cytokine levels, indicating the establishment of immunopathological processes. These findings demonstrate that RNV retains the capacity to induce systemic and neuroinvasive disease under conditions of developmental immune immaturity. Our study provides the first comprehensive *in vivo* characterization of RNV pathogenesis and supports the need to consider this enzootic alphavirus within the broader context of VEEV-complex viruses potentially associated with underrecognized febrile disease in endemic regions, particularly in light of the recent report of a clinically apparent and molecularly confirmed human infection.

## 1 Introduction

The global resurgence of epidemics has renewed attention to arthropod-borne viruses (arboviruses). Nevertheless, many enzootic arboviruses remain poorly characterized, particularly in developing regions where they often circulate silently or cause clinically indistinguishable febrile illness. As a result, their pathogenic potential and contribution to disease burden remain incompletely defined.

Venezuelan equine encephalitis virus (VEEV) belongs to a complex of antigenically related alphaviruses that circulate throughout the Americas (https://talk.ictvonline.org/). Traditionally, VEEV variants have been classified as epidemic/epizootic or enzootic, based on their transmission cycles and association with outbreaks of disease in humans and equines. While epidemic variants are responsible for periodic outbreaks of severe disease, enzootic viruses have long been considered of limited pathogenic relevance based largely on their transmission ecology and apparent lack of association with large epidemics (Guzmán-Terán et al., 2020). However, human exposure to enzootic VEEV strains has been documented, including in urban settings, and infections may range from asymptomatic to mild febrile illness that is clinically indistinguishable from other arboviral infections, such as dengue. Consequently, the burden and pathogenic potential of enzootic VEEV infections are likely underestimated (Aguilar et al., 2011).

In recent years, increasing attention has been paid to enzootic arboviruses that, while not associated with large outbreaks, may cause significant disease under specific ecological or host-related conditions (Guth et al., 2020). Within this context, enzootic viruses of the Venezuelan equine encephalitis virus (VEEV) complex, including Río Negro virus (RNV), have been shown to circulate widely in Argentina and neighboring countries. RNV has been detected in mosquitoes and rodents from multiple Argentine provinces, indicating sustained enzootic transmission and geographic expansion (Contigiani et al., 1999; Mitchell et al., 1985; Pisano et al., 2010, 2012). Serological evidence of RNV infection has also been reported in humans and equines across several regions of Argentina and Uruguay, with equine seroprevalence reaching up to 20% in some areas (Burgueño et al., 2018; Pisano et al., 2013). In addition, bats have been proposed as potential hosts in the natural cycle of RNV, following the detection of viral genomes in oral swabs (Fagre and Kading, 2019; Moreira Marrero et al., 2022). Importantly, the recent report of a clinical apparent and molecularly confirmed human case of RNV infection in Bolivia (Mafayle et al., 2023) challenges the long-standing perception of this virus as clinically insignificant and underscores the need for experimental evaluation of its pathogenic capacity. Altogether, these findings indicate that RNV represents a potential threat to both human and equine health in the Southern Cone of Latin America.

Epizootic strains of Venezuelan equine encephalitis virus (VEEV) have been extensively studied due to their capacity to cause severe outbreaks in equine and human populations. Experimental studies in laboratory mice have shown that these variants replicate systemically, invade the central nervous system, and trigger robust inflammatory responses that contribute to neurological disease (Charles et al., 2001; Jackson et al., 1991; Sharma and Knollmann-Ritschel, 2019; Taylor and Paessler, 2013; Wang et al., 2001). In contrast, the pathogenic potential of natural enzootic VEEV species not belonging to subtype I has been far less explored in animal models. Consequently, the virulence spectrum across enzootic members of VEEV complex remains incompletely defined. Early studies using non-human primates or rodents infected with enzootic strains such as Mucambo virus or Everglades virus reported limited or mild disease following peripheral inoculation, despite evidence of viral neuroinvasion (Coffey et al., 2004; Verlinde, 1968). With regard to Río Negro virus (RNV, subtype VI), early *in vivo* characterizations demonstrated an apparent lack of pathogenicity in immunocompetent adult mice and guinea pigs, even at high inoculation doses (Calisher et al., 1985). However, experimental approaches restricted to adult immunocompetent models may overlook age- or immunity-dependent pathogenic features that emerge under alternative conditions (Cormier et al., 2010; Rivarola et al., 2018; Roth et al., 2020). Consistent with this notion, young mice have been successfully used to reveal disease phenotypes caused by viruses that are otherwise non-pathogenic in adult wild-type animals, including alphaviruses such as Chikungunya virus (Haese et al., 2016).

The aim of the present study is to define the pathogenic profile of RNV in a susceptible murine host, with particular attention to tissue tropism, neuroinvasion, and host inflammatory responses. By simulating the vulnerability of immature immune systems, we seek to expand the currently limited data available for evaluating the pathogenic and epidemic potential of this neglected alphavirus. In the context of ongoing ecological change, including landscape transformation and climate-driven shifts in vector ecology, understanding the pathogenic capacity of enzootic arboviruses such as RNV is increasingly relevant for interpreting febrile disease surveillance data in endemic regions (García-Romero et al., 2023). Of particular concern is the potential involvement of enzootic or epizootic Venezuelan equine encephalitis virus strains in emergent urban transmission cycles, especially given the demonstrated competence of *Aedes aegypti* to transmit both epidemic and enzootic variants of the virus (Ortiz et al., 2008).

## 2 Materials and methods

### 2.1 Virus and mice

The Rio Negro virus strain (RNV, strain F89) used in our study was originally isolated from rodents captured in Formosa province, Argentina, in 1991 (Contigiani et al., 1999), and underwent three passages in suckling mouse brains. This virus was provided by Dr. Marta S. Contigiani from the Laboratory of Arboviruses at the Virology Institute of the Universidad Nacional de Córdoba, Argentina. Working stocks were prepared directly from this material by homogenizing the previously infected mouse brain tissue (10% w/v) in Eagle’s minimum essential medium (MEM) supplemented with 10% fetal bovine serum and 1% gentamicin. Virus titers were determined using a Vero cell plaque assay and expressed as plaque forming units per milliliter (PFU/mL) (Contigiani and Sabattini, 1987). For all experiments, virus inoculations were performed via dorsal subcutaneous injection, administering 10³ PFU per mouse in 0.1 mL of serum-free MEM, prepared by dilution of the original viral stocks. Control groups were inoculated with 0.1 mL of serum-free MEM. C57BL/6 mice were housed in a specific-pathogen-free (SPF) environment, kept in microisolated ventilated cages, and handled by highly trained personnel within a laminar BSL2 flow hood. All animal procedures were approved by the Institutional Committee for the Care and Use of Laboratory Animals (CICUAL) of the Facultad de Ciencias Médicas, Universidad Nacional de Córdoba, under protocol number V-14/2022. All *in vivo* and *in vitro* experiments with RNV were conducted under enhanced biosafety level 2 (BSL-2+) conditions, in compliance with institutional risk assessments and local regulations. Enhanced laboratory practices were applied throughout all procedures to minimize exposure risk.

### 2.2 Survival

To determine the most appropriate age group for pathogenicity studies, we evaluated the susceptibility of C57BL/6 mice of different ages to RNV infection. Male and female mice aged 16 (n = 24), 17 (n = 22), 18 (n = 20), 19 (n= 22), and 20 (n= 20) days were used. For each age group, animals from three different litters were included. After infection, survival was monitored daily for 15 days post-infection (dpi). The confirmation of infection in deceased animals was achieved by detecting infectious viral particles in brain homogenates using Vero cell plaque assays (Contigiani and Sabattini, 1987). Surviving mice were tested for neutralizing antibodies using the plaque reduction neutralization test (PRNT) (Earley et al., 1967) on sera samples (diluted at 1:5 in MEM).

### 2.3 Viremia and viral load in tissues

Viremia was assessed in groups of 18-day-old 5 mice at different time points, expressed as hours post-infection (hpi): 12, 14, 19, 24, 30, 48, 72, and 96. Control groups consisted of three mock-infected (MEM) mice per time point. Blood samples were collected at each time point via submandibular vein puncture. After induction of anesthesia with isoflurane inhalation (2%), euthanasia was carried out via cervical dislocation by trained personnel. Viral loads in serum samples were determined using a standard Vero cell plaque assay and expressed as the logarithm of PFU per milliliter (log PFU/mL).

To evaluate viral dissemination, 27 mice aged 18 days were infected, and three animals were sacrificed daily from day 0 to 9 post-inoculation for tissue collection, including brain, spleen, axillary lymph nodes (near the inoculation site), lungs, pancreas, liver, heart, and thymus. In addition, 3 age-matched uninfected mice were used as controls and sacrificed on day 9 of the experiment. Prior to sacrifice, mice were anesthetized with an intraperitoneal (IP) injection of xylazine (10 mg/kg) and ketamine (100 mg/kg) and perfused transcardially with PBS until the liver was cleared (approximately 4–5 minutes). Approximately half of each tissue sample was weighed, suspended in MEM supplemented with 10% FBS and 1% gentamicin (1/10 w/v), and homogenized using a sterile cold mortar and pestle. The homogenates were clarified by centrifugation at 10,000 x *g* for 30 min, and the supernatants were collected to determine viral loads.

### 2.4 Histology

A fraction of each collected tissue sample was fixed overnight in 10% neutral buffered formalin, embedded in paraffin, sectioned (4 µm), and stained with hematoxylin and eosin (HE) for histopathological examination. Micrographs were taken using a Leica ICC50 E camera attached to a Leica DM500 binocular microscope. To provide a semiquantitative assessment, a scoring system was developed following (Gibson-Corley et al., 2013). Ten randomly selected non-overlapping photomicrographs per section were captured at 10x magnification, and all slides were scored by a single observer. For brain sections, two parameters were scored independently: (1) meningoencephalitic changes (0 = absent; 1 = mild perivascular or meningeal infiltrates; 2 = moderate-to-severe infiltrates with coalescent perivascular cuffing; and (2) perivascular cuffing, expressed as the percentage of positive fields per animal. For lung sections, peribronchiolar/alveolar inflammation was scored per field (0 = absent; 1 = mild; 2 = moderate; 3 = severe). In addition, tissue remodeling was assessed using a grid-based approach in which each photomicrograph was divided into quadrants, and the proportion of affected quadrants was expressed as percentage involvement per image.

### 2.5 Spleen immunofluorescence

Spleens from 2 control and 2 RNV-infected mice were collected at 2, 4 and 6 dpi and immediately flash-frozen in liquid nitrogen. Cryosections (7 µm) were fixed in cold acetone for 10 min, air-dried at room temperature (RT), and stored at -80°C until further use. Prior to staining, slides were rehydrated in Tris buffer and incubated for 30 min at 25°C in blocking solution containing 10% normal mouse serum diluted in Tris buffer (Beccaria et al., 2018). After blocking, slides were incubated for 50 min at 25°C with different combinations of the following anti-mouse Abs: PE-labeled anti-CD8a (Cat# 12-0081-83, clone 53-6.7, dilution 1/100), APC-labeled anti-B220 (Cat# 17-0452-83, clone RA3-6B2, dilution 1/200) from eBiosciences, and AlexaFluor 488-labeled anti-CD169 (Cat# 142419, clone 3D6.112, dilution 1/150) from BioLegend. Slides were mounted using FluorSave reagent (Merck Millipore), and images were acquired with an Olympus FV1000 confocal microscope. To ensure the robustness of the qualitative analysis, at least five sections spanning different splenic regions were examined per organ, with multiple follicles analyzed per section to confirm consistent histological patterns. Image analysis was performed using ImageJ/Fiji software (version 1.52e; NIH, USA) (Schneider et al., 2012).

### 2.6 Analysis of neurodegeneration

Three infected mice were sacrificed at 2 dpi (n=3, with no signs of illness) or at 8 dpi (n=3, with neurological signs). Chloral hydrate (0.3g/Kg) was injected intraperitoneally (IP) for anesthesia and each mouse was transcardially perfused, rinsed with glucose (0.4%), sucrose (0.8%), and sodium chloride (0.8%), and fixed with 4% paraformaldehyde (PFA) in 0.2 M borate buffer (pH 7.4). After immersion in 30% sucrose, brains were cut in frontal and sagittal sections (40µm) using a cryostat. A subset of sections was reserved for immunohistochemical analyses. Neurodegeneration was assessed in brain tissue using the amino-cupric-silver (A-Cu-Ag) staining technique, performed according to the method described by de Olmos et al (De Olmos et al., 1994). Briefly, brain sections were rinsed with Milli-Q water and incubated in a silver nitrate solution at 50°C (pre-impregnation). Once back at room temperature, sections were rinsed with acetone and transferred to a concentrate silver diamine solution for 40 min. They were immersed in a formaldehyde/citric acid solution for 25 min and the reaction was stopped in 0.5% acetic acid. A two steps bleaching was performed to remove any non-specific deposit of silver, first in 6% potassium ferricyanide, washed with Milli-Q water, then transferred to 0.06% potassium permanganate for 20s. After washing, stabilization was achieved in 2% sodium thiosulphate, then the sections were washed again, transferred to a fixer solution for 1 min, and mounted on slides. Once dried, slides were cleared by immersion in xylene for 10 min before coverslipping. Images were obtained in an optic microscope (OlympusCX40) equipped with a Zeiss AxioCam ERc5s camera. Neuronal degeneration was evaluated qualitatively on cortical structures and striatum of infected mice. Sections of uninfected mice were analyzed as controls. Anatomical localization and brain mapping were performed according to the mouse brain stereotaxic atlas by Franklin and Paxinos (2007). To standardize the evaluation of RNV-induced damage, neurodegeneration was categorized into three morphological types based on argyrophilic staining patterns as described in previous reports (Bueno et al., 2003; De Olmos et al., 1981): (1) “apoptotic-like degeneration” (Ap), characterized by granular deposits in the soma and surrounded by granular terminal-like deposits; (2) “terminal degeneration” (TND), identified as dense accumulations of argyrophilic particles in axon terminals and synaptic endings independent of parent cell body death; (3) “somatodendritic degeneration”, defined by argyrophilic somata and dendrites, showing a dark, smoothly contoured profile. The severity of damage in each brain region was assessed using a semi-quantitative scale: (+++) severe/widespread damage, (++) moderate damage, (+) mild/scattered lesions, and (-) no detectable damage. Sections from uninfected mice were analyzed as controls.

### 2.7 Brain immunohistochemistry

Brain sections (40 µm), obtained from the same perfused and cryoprotected brains described above for neurodegeneration analysis, were processed for free-floating immunofluorescence in 6-well plates under constant agitation, following standard protocols. Briefly, 10–15 sections per mouse were washed three times for 5 min each in PBS. Sections were then blocked and permeabilized in PBS containing 0.3% Triton X-100 and 3% FBS for 2 hours at RT. Primary antibody incubation was performed for 24 h at 4°C under agitation, using rabbit polyclonal anti-glial fibrillary acidic protein (GFAP; 1:500; cat. no. G3893, Sigma-Aldrich) diluted in blocking solution (PBS with 1% FBS and 0.3% Triton X-100). After washing, sections were incubated for 1 hour at RT with Alexa Fluor 488-conjugated donkey anti-rabbit IgG (1:1000; cat. no. A21206, Invitrogen), followed by counterstaining with DAPI in PBS for 15 min. Finally, sections were washed three times in PBS, mounted on gelatin-coated slides with Mowiol 4-88 (Sigma-Aldrich), and allowed to dry overnight at room temperature. Images were acquired using a confocal fluorescence microscope (Olympus FV1200, Japan). GFAP immunoreactivity was quantified using Fiji/ImageJ software (version 1.52e; NIH, USA). For each animal, two non-overlapping fields from the hippocampal region were analyzed in sagittal brain sections. Images were acquired under identical exposure settings and converted to 8-bit grayscale. Background subtraction was performed using a rolling ball radius of 50 pixels. A consistent threshold (Triangle method) was applied to all images to identify GFAP-positive signal. The GFAP-positive area was measured and expressed as a percentage of the total analyzed area (% GFAP^+^ area). The mean value per animal was calculated from the analyzed fields and used for statistical analysis.

### 2.8 Detection of inflammatory cytokines

Two groups of three infected mice were euthanized at 2 dpi (first group) or immediately after the onset of neurological symptoms (6 dpi). An uninfected control group (n=3) was processed in parallel. Brains were collected, flash-frozen in liquid nitrogen, and stored at -80°C. After weighing and thawing, tissues were minced using two scalpels and processed according to the protein isolation method described by Amsen et al (Amsen et al., 2009), keeping all samples on ice throughout the procedure. Briefly, tissues were homogenized using a tissue mixer (Janke & Kunkel) in a lysis buffer lacking SDS. Lysates were then sonicated using a probe sonicator (five 30-s pulses with 30-s intervals) and centrifuged at 14,000 × *g* for 20 min at 4°C. Supernatants were collected and cytokine levels were measured using a multiplex Cytometric Bead Array (CBA) system, following the manufacturer’s instructions (Mouse Inflammation Kit, BD Biosciences). Standard and test samples (infected and control groups) were analyzed using an Accuri C6 flow cytometer (BD).

### 2.9 Statistical analyses

Data were analyzed using GraphPad Prism (version 8.0.2). Normality was assessed with the Shapiro-Wilk test, followed by a one-way ANOVA to compare mean values across experimental groups or time points. *Post-hoc* comparisons were performed using Tukey’s multiple comparisons test, with statistical significance set at p < 0.05. Survival data were analyzed using Kaplan–Meier survival curves. Deaths were coded as events (value = 1), and animals surviving until the end of the observation period were treated as censored data (value = 0). Differences among age groups were assessed using the log-rank (Mantel–Cox) test, and age-dependent trends in survival were evaluated using the log-rank test for trend. Median survival times (in dpi) were calculated for each age group. For viremia, viral titers were directly analyzed without prior adjustment and are presented as the logarithm of plaque-forming units per milliliter (log PFU/mL). For organotropism, viral titers in tissue homogenates collected at different dpi were analyzed using the same statistical approach to evaluate differences among time points. For GFAP immunofluorescence analysis, the percentage of GFAP-positive area (% GFAP^+^ area) was calculated for each field and averaged per animal. These values were then used for statistical comparisons among experimental groups using one-way ANOVA followed by Tukey’s *post hoc* test, as described above. For cytokine quantification by CBA, raw cytometry data were first processed using a nonlinear regression 3PL curve fit to obtain interpolated values, which were then subjected to statistical analysis as described.

## 3 Results

### 3.1 RNV infection causes age-dependent disease and mortality in mice

**Figure 1:**
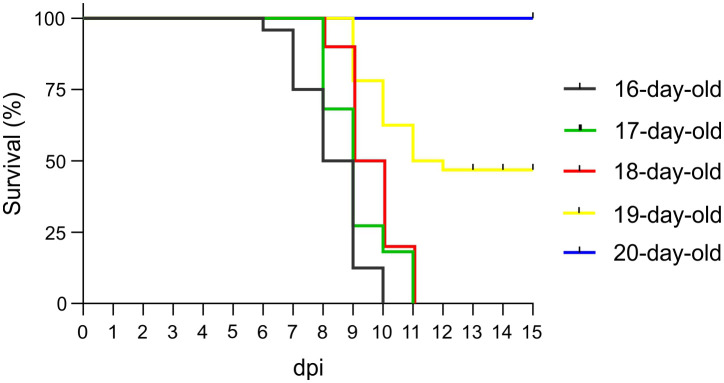
Age-dependent survival following RNV infection in C57BL/6 mice. Infant mice aged 16–20 days were infected and monitored daily for survival over a 15-day period (dpi, days post-infection). Each curve represents pooled data from three litters per age group (n = 20–24 per group).

To determine an appropriate murine model for studying RNV pathogenesis, we first assessed age-dependent susceptibility in infant C57BL/6 mice. Animals aged 16 to 20 days were subcutaneously infected and monitored for signs of illness, mortality, and evidence of infection. Marked differences in survival were observed among age groups (**Figure 1**). The log-rank (Mantel-Cox) test revealed highly significant differences between survival curves (χ² = 86.49, df = 4, p < 0.0001), indicating that survival probability strongly depended on age at the time of infection. The log-rank test for trend also showed a significant linear association (χ² = 57.83, df = 1, p < 0.0001), consistent with a progressive increase in survival with age. All 20-day-old mice survived without apparent illness, unlike younger groups that showed 100% mortality at 16–18 days of age and early clinical signs (ruffled fur, lethargy) appearing 2–3 dpi (**Figure 1**). In these younger animals, the disease evolved into ataxia and paralysis before culminating in death. Infection was confirmed in all deceased mice by detecting infectious viral particles in brain homogenates, whereas surviving animals developed neutralizing antibodies against RNV, with titers exceeding 1:20.

The survival curves clearly illustrate a sharp decline in susceptibility to RNV infection between 18 and 20 days of age. The 18-day-old group, being the oldest cohort to exhibit complete mortality, was therefore selected for subsequent pathogenicity experiments.

### 3.2 RNV infection in infant mice results in systemic dissemination and broad organ tropism

**Figure 2:**
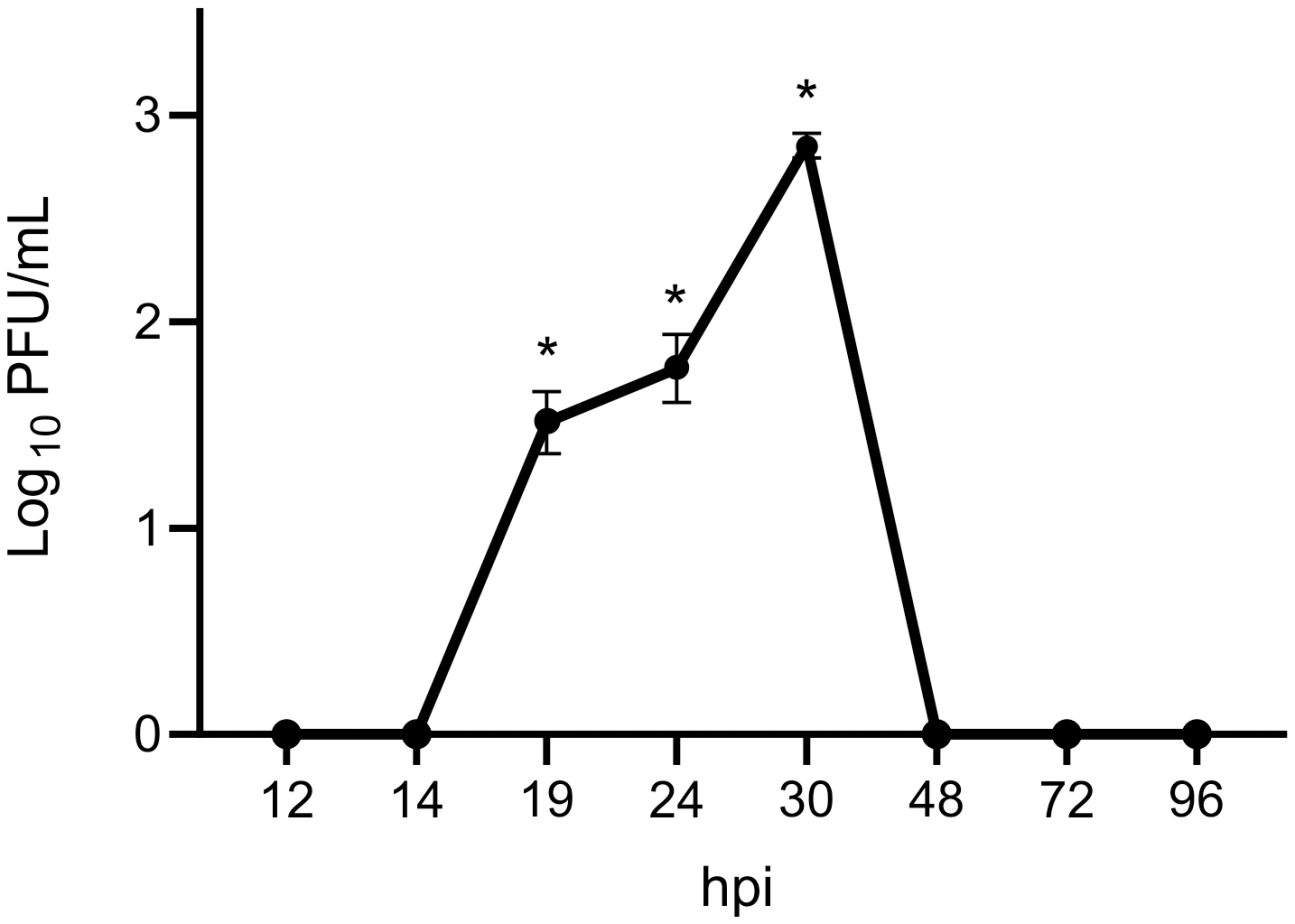
RNV infection results in transient viremia in infant mice. Viral titers in serum samples are expressed as log PFU/mL at different hours post-infection (hpi). Data points represent the mean of five mice per time point, with error bars indicating standard deviations. Asterisks (*) denote statistically significant increases in mean viral titers between consecutive time points (*p* < 0.05, one-way ANOVA followed by Tukey’s multiple comparisons test).

Viremia was detected between 14- and 48- hpi, with a peak of 2.85 ± 0.03 log_10_ PFU/mL at 30 hpi. No secondary viremia was detected in blood samples collected daily up to 10 dpi, and no infectious virus was detected in serum from mock-infected control mice at any time point (**Figure 2**).

**Figure 3:**
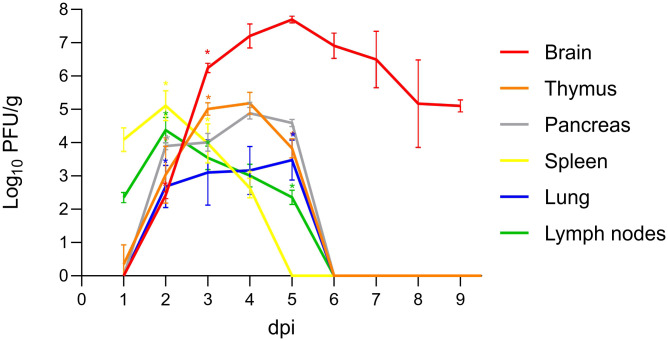
RNV infection leads to systemic dissemination and broad tissue tropism. Viral titers (log PFU/g) were measured in various organs at different dpi using Vero cell plaque assays. Data points represent the mean viral load per gram of tissue from three mice per time point, with error bars indicating standard deviations. The detection limit of the plaque assay was 1 Log_10_ PFU/g. Asterisks (*) denote statistically significant differences in viral titers between consecutive time points within each organ (*p* < 0.05, one-way ANOVA followed by Tukey’s multiple comparisons test).

To further investigate the systemic impact of infection, we analyzed the distribution of viral particles across multiple organs. No infectious virus was detected in the heart or liver, whereas significant viral replication was observed in the spleen, lymph nodes, thymus, pancreas, lung, and brain (**Figure 3**). Viral dissemination occurred rapidly, with detectable loads in lymphatic tissues as early as 24 hpi. Peak mean viral titers occurred on day 2 post-infection (pi) in the spleen (5.11 ± 0.45 log PFU/g) and lymph nodes (4.38 ± 0.38), at 4 dpi in the pancreas (4.88 ± 0.17), lungs (3.16 ± 0.72), and thymus (5.18 ± 0.33), and at 5 dpi in the brain (7.70 ± 0.10). A one-way ANOVA revealed significant differences in viral titers across tissues (F = 40, p < 0.05). *Post hoc* comparisons showed that viral loads in the brain were significantly higher than in all other tissues (p < 0.05). Moreover, brain infection persisted the longest, extending up to day 9 pi, coinciding with the terminal phase of neurological disease. Neurological symptoms first became evident from day 6 pi onward, shortly after peak viral titers were detected, reflecting a temporal association between viral replication in the CNS and the onset of neurological manifestations.

### 3.3 RNV induces histopathological alterations in infected tissues

**Figure 4:**
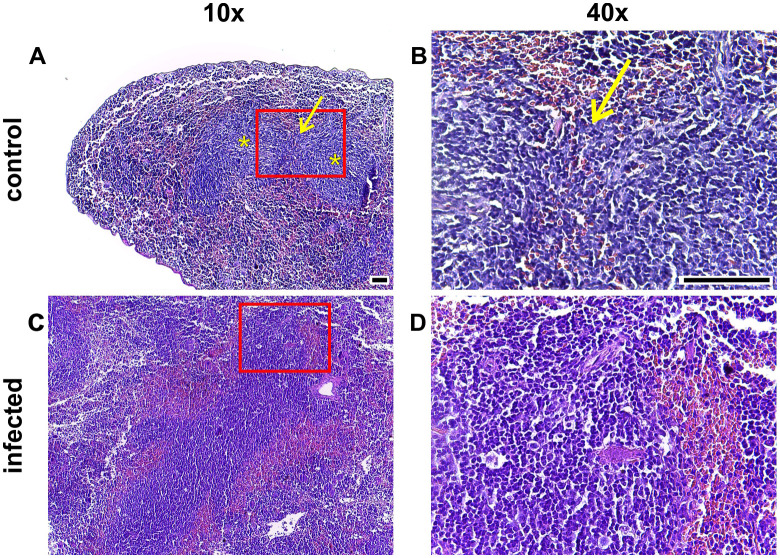
RNV infection induces alterations in splenic architecture. Representative HE-stained spleen sections at 10x (left panels) and 40x (right panels) magnification. **(A, B)** Mock-infected control mouse showing preserved splenic architecture, with well-defined white pulp follicles (yellow asterisks) and clearly demarcated interfaces between white and red pulp (yellow arrows). **(C, D)** Spleen from a RNV-infected mouse at 6 dpi. Magnification: 10x **(A, C)** and 40x **(B, D)**. Scale bars: 500 µm (10x and 40x). Red boxes in 10x panels indicate the region shown at higher magnification on the right. Images are representative of one out of 3 mice per group.

We assessed the effects of RNV infection on selected tissues at different dpi, corresponding to periods of active viral replication. In HE-stained spleen sections from infected mice, changes suggestive of architectural disorganization were observed by 6 dpi, with the boundaries between white and red pulp appearing less clearly defined compared with the well-demarcated regions observed in control animals (**Figures 4A, B**, arrows) (**Figures 4C, D**).

**Figure 5:**
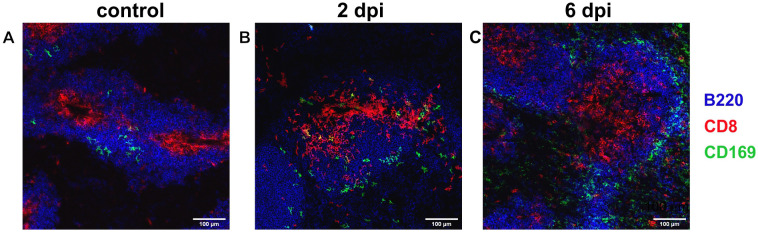
Disruption of splenic immune architecture in RNV-infected mice. Representative images of spleen sections from uninfected control mice **(A)** and RNV-infected mice at 2 **(B)** and 6 **(C)** days post-infection (dpi), stained with anti B220 (blue, B cells), anti CD8 (red, T cells), and anti CD169 (green, macrophages). Images were acquired with an Olympus FV1000 confocal microscope at 20x magnification. Scale bars: 100 µm. Findings are representative of multiple non-consecutive sections analyzed per animal (n = 2 mice per group/time point).

To further investigate the splenic alterations revealed by HE staining, we performed immunofluorescence to evaluate the organization of immune cell compartments. **Figure 5A** shows a representative spleen section from uninfected control mice, displaying a well-organized structure with clearly defined B-cell (B220^+^, in blue) and CD8^+^ T-cell in red) zones, together with a discrete presence of CD169^+^ metallophilic macrophages (in green) (). In contrast, infected mice exhibited a severe and progressive disorganization of the lymphoid microenvironment. This pattern was consistently observed across all analyzed sections, where as early as 2 dpi, B-cell follicle boundaries became diffuse, with notable infiltration of CD8^+^ T cells into B220^+^ areas (**Figure 5B**). By 6 dpi, this disorganization was markedly exacerbated, with widespread loss of compartmentalization and increased dispersion of both CD8^+^ T cells and CD169^+^ metallophilic macrophages surrounding B cell follicles (**Figure 5C**).

**Figure 6:**
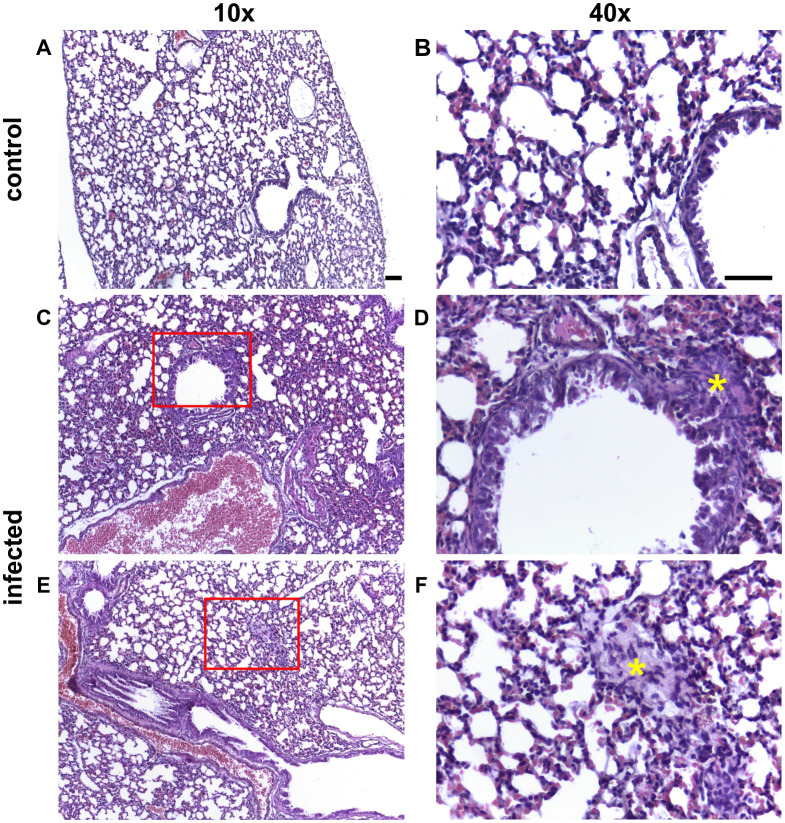
RNV infection induces inflammatory pathology in the lungs. HE-stained lung sections (4 µm) from uninfected control mice **(A, B)** and RNV-infected mice at 8 days post-infection **(C–F)**. Panels **(A, C, E)** were captured at 10x magnification; panels **(B, D, F)** at 40x magnification. Magnification: 10x **(A, C, E)** and 40x **(B, D, F)**. Scale bars: 500 µm (10x and 40x). Red rectangles in the 10x images indicate the areas shown at higher magnification in the corresponding 40x magnification. Photographs are representative of one out of 3 mice at each time point.

HE-stained lung sections from uninfected mice showed preserved alveolar architecture, characterized by thin septa and sparse interstitial cellularity (**Figures 6A, B**). In contrast, infected mice analyzed at 8 dpi displayed pronounced thickening of alveolar septa and a marked increase in interstitial cellularity, consistent with inflammation and tissue remodeling (**Figures 6C, E**, rectangles; **Figures 6D, F**, asterisks). Grid-based analysis of 10x photomicrographs from infected mice revealed that 23-62% of analyzed quadrants per field showed evidence of peribronchiolar or alveolar inflammatory infiltrates, whereas 0-8% showed areas compatible with tissue remodeling and septal thickening. These findings are consistent with a mild-to-moderate interstitial pneumonia pattern and confirm that the pulmonary inflammation was not a focal incidental finding, but rather involved a considerable proportion of the lung parenchyma.

**Figure 7:**
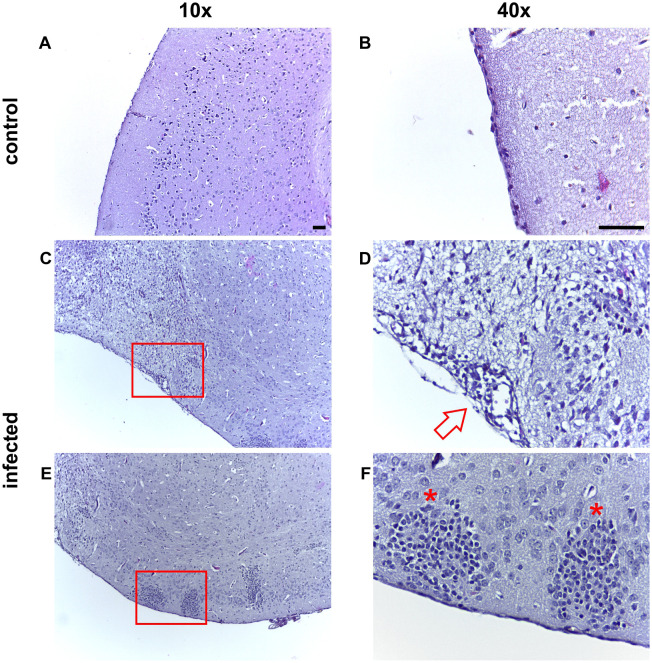
RNV infection results in histopathological changes in the brain. Panels **(A, B)** correspond to uninfected control mice. Panels **(C–F)** show brains from infected mice at 8 days post-infection. Magnification: 10x **(A, C, E)** and 40x **(B, D, F)**. Red rectangles in the 10x images indicate the areas shown at higher magnification in the corresponding 40x magnification Scale bars: 500 µm (10x and 40x). Photographs are representative of one out of 3 mice at each time point.

Consistent with the neurological symptoms observed in infected animals, clear pathological alterations were also detected in brain sections from RNV-infected mice, which became more pronounced at later stages of infection. HE-stained brain sections from infected mice revealed localized inflammatory processes in the meninges, characterized by edema, meningeal thickening, and immune cell infiltration (**Figures 7C**, rectangle; **Figures 7D**, arrow). In addition, multiple foci of inflammatory cell infiltration were found throughout the cortex, frequently coalescing into discrete clusters (**Figures 7E**, rectangle; **Figures 7F**, asterisks).

**Figure 8:**
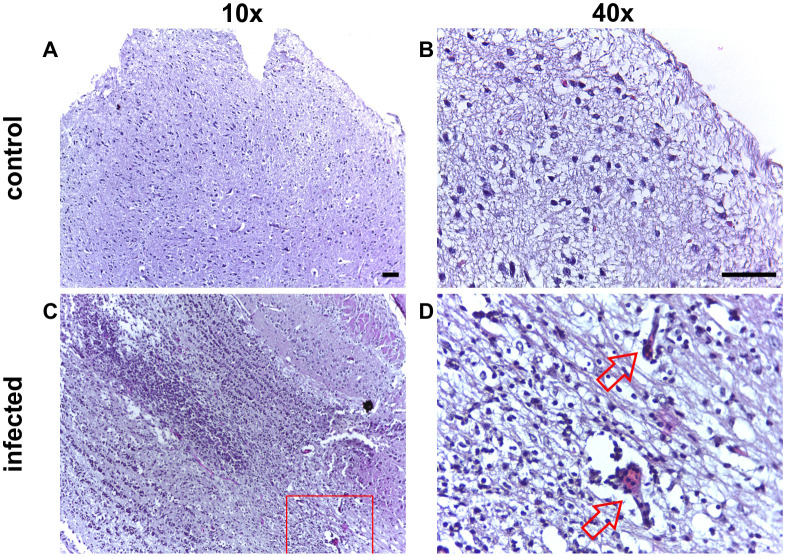
Histopathological alterations in the olfactory bulb following RNV infection. Panels **(A, B)** correspond to uninfected controls. Panels **(C, D)** show infected mice at 8 days post-infection. Sections were stained with HE and cut at 4 µm thickness. Magnification: 10x **(A, C)** and 40x **(B, D)**. The red rectangle in the 10x image indicates the area shown at higher magnification in the corresponding 40x panel. Scale bars: 500 µm (10x and 40x). Images are representative of three mice per group.

To complement the qualitative observations described above, we applied a semiquantitative scoring system to HE-stained sections of brains. Meningoencephalitic alterations were detected in 83-100% of analyzed fields across symptomatic animals, whereas perivascular cuffing was observed in 60-83% of analyzed fields. These inflammatory alterations were consistently associated with moderate-to-severe meningeal and perivascular infiltrates, frequently forming coalescent perivascular cuffs. In addition, vascular congestion with intraluminal nucleated cells was observed in multiple sections (**Figures 8C, D**, arrows), further supporting the presence of marked neuroinflammatory alterations.

### 3.4 RNV infection triggers neurodegeneration and glial activation

**Figure 9:**
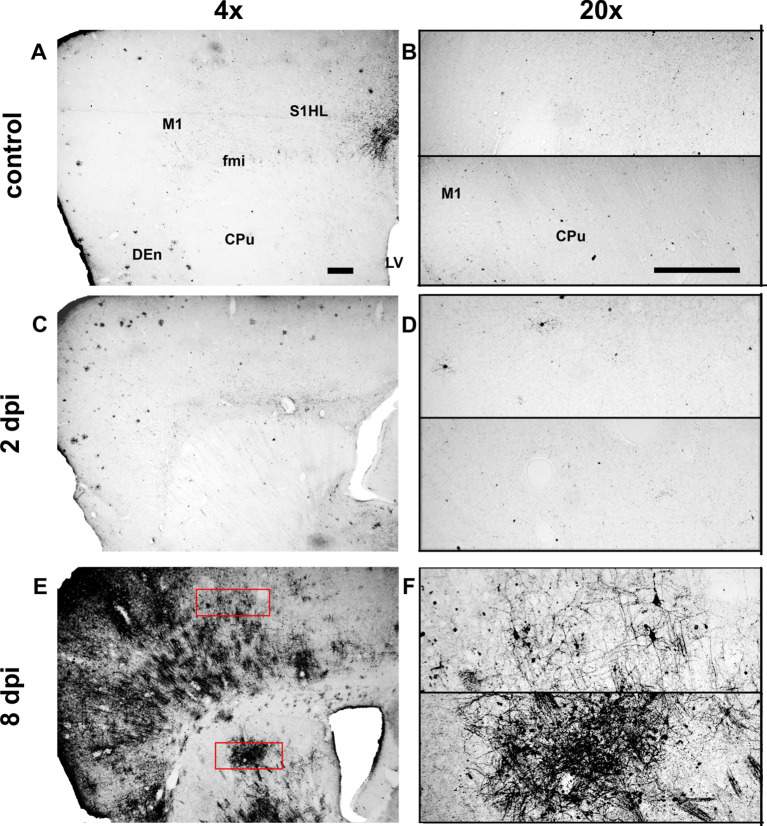
RNV infection induces neurodegeneration in the brain. Representative sagittal brain sections stained with the amino-cupric-silver (A-Cu-Ag) technique. Panels **(A, B)** show an uninfected age-matched control mouse. Panels **(C, D)** correspond to mice infected with RNV and euthanized at 2 days post-infection (dpi). Panels **(E, F)** display brain sections from infected mice at 8 dpi. Degenerative foci appear as dark silver-stained structures. Left panels: 4x magnification; right panels: 20x magnification. M1: primary motor cortex; S1HL: somatosensory cortex (hind limb area); CPu: caudate-putamen; fmi: forceps minor of corpus callosum; DEn: dorsal endopiriform nucleus; LV: lateral ventricle. Scale bars: 500 µm (4x) and 100 µm (20x). Images are representative of 3 mice per group.

We subsequently investigated whether RNV infection could induce neurodegeneration in specific brain regions, both prior to the onset of neurological symptoms (2 dpi) and at terminal stages of disease (8 dpi). **Figure 9** shows sagittal brain sections stained with the A-Cu-Ag technique, highlighting the progression of degeneration. The distribution and morphological categorization of these findings (graded as -, +, ++, and +++) are summarized in **Table 1**. As a reference, uninfected mice displayed only isolated foci of physiological cell death, likely associated with normal postnatal development (**Figures 9A, B**). At 2 dpi, infected mice already harbored high viral loads in the brain (exceeding 6 Log PFU/g), but only sparse silver-stained foci were observed (**Figures 9C, D**). Notably, at this early stage (2dpi), argyrophilic markers were detected predominantly in the olfactory bulb and hippocampus, displaying mainly apoptotic-like morphology. In contrast, brains from symptomatic animals at 8 dpi exhibited more extensive somatodendritic and terminal degeneration, prominently clustered around blood vessels (**Figures 9E, F**). As detailed in **Table 1**, this degeneration was particularly severe (+++) in cortical structures and the cerebellum, characterized by extensive terminal and somatodendritic neurodegeneration, which correlates with the onset of severe neurological signs.

**Table 1:** Morphological classification and severity of RNV-induced neuronal degeneration in mouse brains.

Brain region	Days post infection (dpi)	Apoptotic-like degeneration	Somatodendritic degeneration	Terminal degeneration
Olfactory bulb	2	++	–	–
	8	++	–	–
Cortical structures	2	–	–	–
	8	+	+++	++
Hippocampus	2	+	–	–
	8	+	–	–
Caudate putamen	2	–	–	–
	8	–	+	++
Thalamus	2	–	–	–
	8	–	–	–
Hypothalamus	2	–	–	–
	8	–	–	–
Corpus callosum	2	–	–	–
	8	–	+	+
Cerebellum	2	–	–	–
	8		++	+++

+++ severe damage, when the entire area of the brain showed degeneration.

++ moderate damage, when only a part of the area was affected.

+ scattered lesions, with reduced degeneration.

- no damage at all.

**Figure 10:**
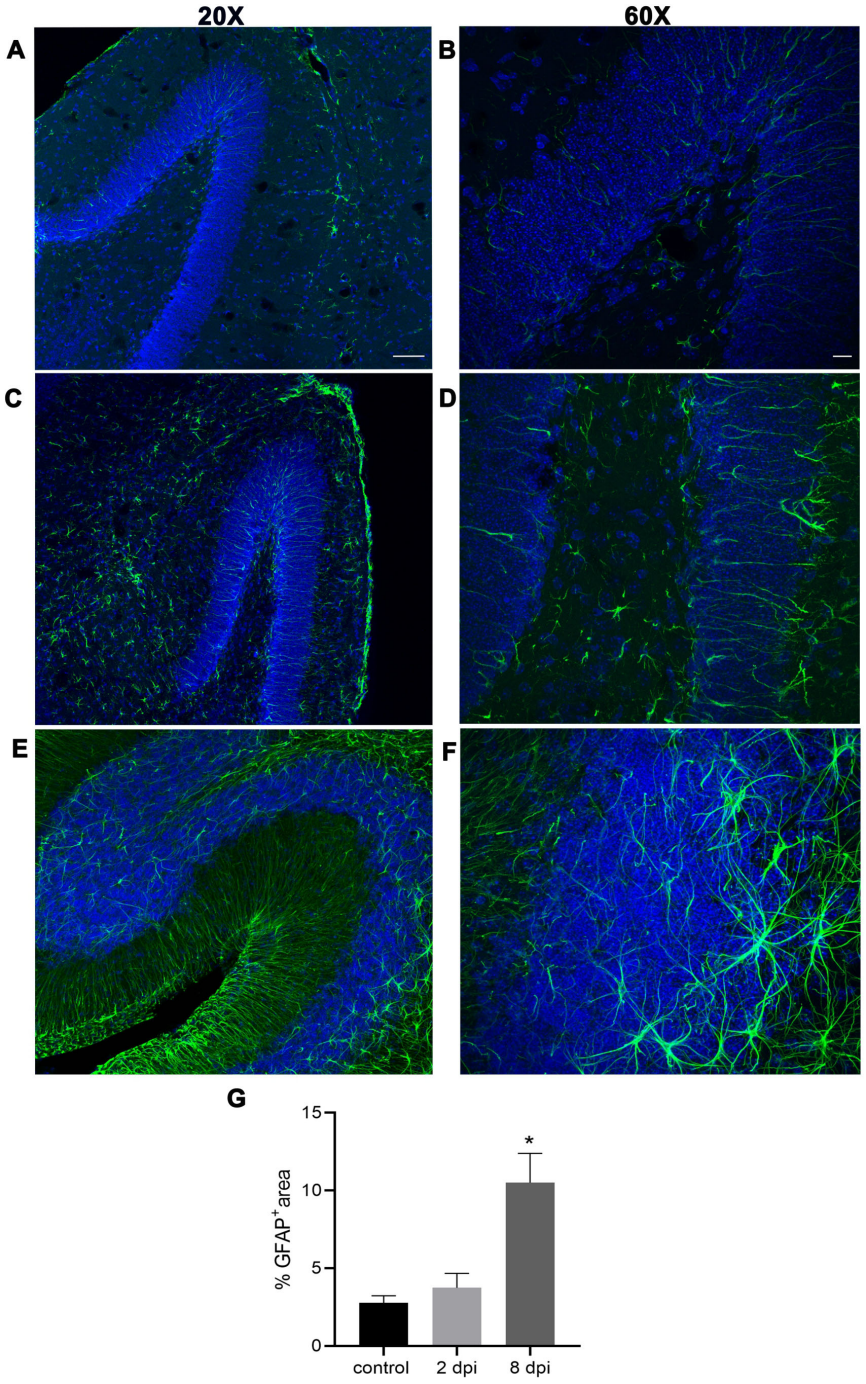
Astrocyte activation in the hippocampus during RNV infection. Immunofluorescent staining of sagittal brain sections at the hippocampal level using anti-GFAP (green) and DAPI (blue). Panels **(A, B)** correspond to uninfected controls. Panels **(C, D)** show infected mice at 2 days post-infection (dpi). Panels **(E, F)** show infected mice at 8 dpi. Left panels: 20x magnification; right panels: 60x magnification. Scale bars: 100 µm (20x) and 20 µm (60x). Images are representative of 3 mice per group. Panel **(G)** shows the quantification of GFAP-positive area among the groups, expressed as percentage of total area (% GFAP^+^ area). Data are presented as mean ± SEM (n = 3). Statistical analysis was performed using one-way ANOVA followed by Tukey’s *post hoc* test. *p < 0.05 vs. control.

To further investigate the neuroinflammatory response to RNV infection, we performed an analysis of astrocyte activation using anti-GFAP staining on brain sections. The staining was performed on coronal sections encompassing the entire brain, and GFAP signal alterations were observed in multiple regions, including the hippocampus, cerebral cortex, and cerebellum. However, for consistency and image clarity, representative visualization was focused on the hippocampal region, where staining was most robust and reproducible across samples. As shown in **Figure 10**, control mice (**Figures 10A–C**) exhibited sparse and finely branched GFAP^+^ astrocytes, mostly confined to specific hippocampal layers. In contrast, infected mice (**Figures 10D–F**) showed progressive increases in GFAP signal intensity and astrocyte hypertrophy. At 2 dpi, GFAP^+^ processes appeared more numerous and thicker, suggesting early astrocyte activation. These changes were markedly intensified at 8 dpi, characterized by dense GFAP immunoreactivity and enlarged astrocytic processes, consistent with a robust astrogliosis. Quantitative analysis confirmed a significant increase in GFAP expression at 8 dpi (**Figure 10G**). While the temporal and spatial overlap between gliosis and neuronal injury remains to be fully resolved, the presence of marked astrocyte activation in multiple brain areas supports a widespread neuroinflammatory response to RNV infection.

### 3.5 RNV induces pro-inflammatory cytokines in the brain

**Figure 11:**
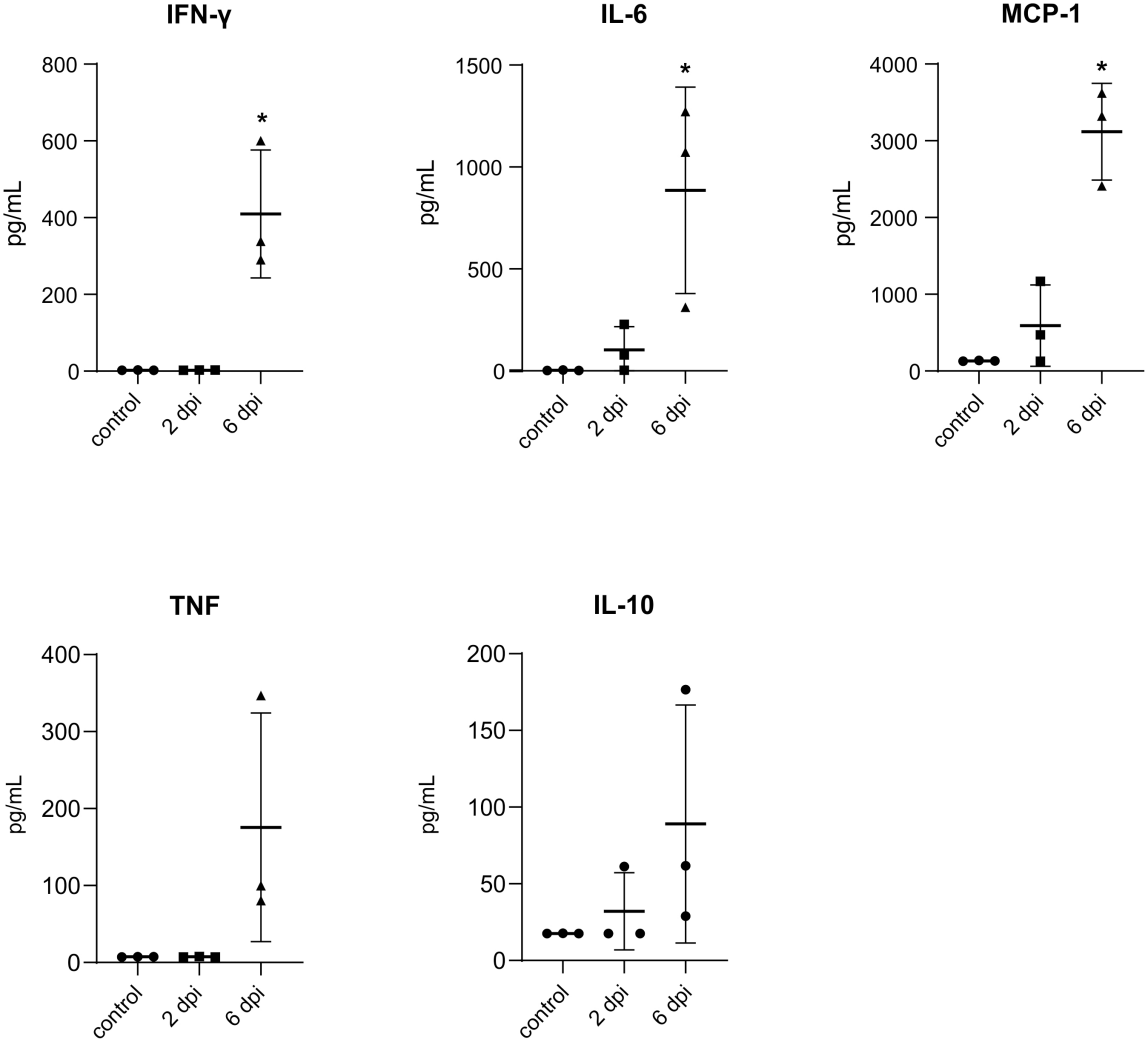
RNV infection induces inflammatory cytokine responses in brain tissue. Levels of IFN-γ, IL-6, MCP-1, TNF, and IL-10 were quantified using a multiplex Cytometric Bead Array (CBA) system. Experimental groups included uninfected controls (n=3), mice at 2 dpi (n=3), and at 6 dpi (n=3). Data are presented as individual values with group means ± SEM. Statistical comparisons were performed using one-way ANOVA followed by Tukey’s multiple comparisons test. *p < 0.05 vs. control. For IL-10 and TNF, no significant differences were found (*p* = 0.2255 and *p* = 0.0841, respectively). These results are from a single experiment using three animals per group.

We next sought to determine whether this glial response was associated with increased expression of inflammatory mediators. To this end, we quantified inflammatory cytokines in the brain using a commercial CBA. A panel of six cytokines was analyzed (IL-6, IL-10, MCP-1, IFN-γ, TNF, IL-12p70) in brains collected at 2 dpi and at 6 dpi. As shown in **Figure 11**, symptomatic animals exhibited significantly elevated levels of IFN-γ, IL-6, and MCP-1 compared to both control and 2 dpi groups. Although no statistically significant differences were detected for TNF or IL-10, an increased dispersion of values was observed in symptomatic animals. IL-12p70 levels remained undetectable across experimental groups. These results indicate that the onset of neurological symptoms is associated with a robust local induction of key pro-inflammatory mediators in the brain.

## 4 Discussion

In general, animals infected with enzootic VEEV subtypes do not develop detectable viremia or disease (Weaver et al., 2004), and virulent phenotypes in epizootic strains have been associated with specific sequences variants in the viral envelope glycoproteins (Greene et al., 2005). Although RNV has not been associated with large outbreaks of acute illness, its silent circulation may represent an underrecognized component of arboviral disease dynamics in regions where enzootic transmission networks persist. The recently reported case of RNV-associated illness in an Argentine agricultural worker in Bolivia (Mafayle et al., 2023) highlights the potential of this virus to cause disease in humans, especially among those whose daily activities bring them into contact with disrupted transmission ecologies shaped by anthropogenic pressures. The reported clinical manifestations included fever, chills, headache, nausea, arthralgia, myalgia, thoracic pain, back pain, hyperemia, and bibasilar crackles, although neurological symptoms were not described. Importantly, the reported human case did not involve a host with known conditions of increased immunological susceptibility comparable to those represented in our experimental model, which may partially account for the differences in clinical presentation.

In this context, we describe RNV pathogenesis in a susceptible mouse model. While previous studies reported no disease in adult immunocompetent laboratory rodents, we observed clear signs of progressive neurological illness and uniform lethality in 18-day-old infant wild-type mice. This pre-weaning stage represents a window of biological vulnerability that reflects the clinical observations in human VEEV-complex outbreaks, where children and older adults are disproportionately at risk for severe neurological disease, including convulsions, coma, and death (Dietz et al., 1979; Rivas et al., 1997). Importantly, the use of wild-type mice at this stage allowed us to investigate disease progression under conditions of natural immune immaturity, without the need for immunosuppressed or genetically modified models. Age-dependent susceptibility has been described in several viral models, including both arthritogenic and encephalitic alphaviruses, which tend to show increased tissue invasion and severity in younger hosts (Couderc et al., 2008; Griffin et al., 1994; Kundin et al., 1966; Labrada et al., 2002; Manangeeswaran et al., 2020; Ryman et al., 2007).

To further characterize RNV pathogenesis in this model, we analyzed the profile and implications of viremia. Following infection, infant mice exhibited a short-lived viremic phase, reaching a relatively low peak viral titer of 10^3^ PFU/mL (**Figure 2**). Although no data are currently available for viremias induced by other strains within VEEV subtype VI, reports on related variants offer useful comparisons. Ludwig et al. (2001) reported similarly brief and low-level viremias (~200 PFU/mL) in mice infected with attenuated vaccine strains, in contrast to epidemic strains such as Trinidad Donkey, which reached titers of ~10^6^ PFU/mL. Importantly, low viremia does not preclude transmission: Culex mosquitoes can acquire enzootic VEEV strains from vertebrate hosts with titers as low as 10³–10^4^ PFU/mL (Cupp et al., 1986, 1981), and comparable levels have been observed in natural reservoir hosts experimentally infected with subtype ID strains. Similar viremia levels were observed by Carrara et al. in *Proechimys chrysaeolus* spiny rats (mean peak: 3.3 log10 PFU/mL) experimentally infected with a subtype ID strain of VEEV (Carrara et al., 2005; Smith et al., 2006). In those settings, systemic infection occurred without overt disease. By contrast, in our model, a similarly modest and transient viremia was sufficient to enable widespread tissue dissemination, including efficient neuroinvasion and fatal encephalitis in infant mice, despite the absence of sustained secondary viremia.

To understand how RNV progresses following its brief viremic phase, we examined its distribution across multiple tissues. In our study, RNV reached high viral loads in spleens and lymph nodes as early as day 1 post-infection. Similar early replication in lymphoid tissues has been reported in adult mice infected with wild-type epizootic VEEV, where these compartments -draining lymph nodes and spleen- likely serve as primary sites of viral replication as early as 6 hpi (Sharma and Knollmann-Ritschel, 2019). As a major lymphoid filter and site of intense viral replication, the spleen may provide a microenvironment conducive to early diversification of viral populations (Domingo et al., 2012). While viral loads in peripheral organs declined after peaking, infection in the brain persisted throughout the course of neurological disease. The sustained presence of RNV in the brain, despite clearance from the bloodstream, suggests that CNS invasion occurs early during infection. Notably, the absence of detectable virus in other peripheral sites, such as the heart and liver, contrasts with the high titers observed in the affected tissues. While we cannot rule out the presence of viral RNA or low-level transient replication in these organs, our results indicate that they do not support robust productive infection under the conditions of this model. This organ-specific pattern of infection, particularly the sustained neurotropism, prompted us to further examine the histopathological alterations underlying disease progression.

Here, we report that RNV induced splenic architectural disorganization. The altered spatial distribution of B220^+^ B cells, CD8^+^ T cells, and CD169^+^ macrophages, was strikingly consistent across all examined specimens, evidencing inflammation-associated remodeling (**Figure 5**). Proper spatial segregation of immune cell subsets is essential for adaptive immune responses, including antigen presentation and germinal center formation (Lewis et al., 2019); thus, the extensive disruption observed here could be linked to impaired humoral immunity and viral persistence (Benedict et al., 2006; Kuka and Iannacone, 2018). In our model, the severity and consistency of this splenic architectural disruption may help explain the failure of infant mice to mount an effective immune response, ultimately permitting uncontrolled viral dissemination, as evidenced by the development of pronounced pulmonary pathology. Future studies specifically focused on the cellular and immunological alterations associated with RNV infection should incorporate quantitative approaches, such as flow cytometry or other cell-specific quantification strategies, to further characterize the cellular populations potentially involved in this process.

Although moderate to severe interstitial pneumonia -characterized by infiltration of alveolar septa by neutrophils, lymphocytes, and macrophages- has been described in human VEEV infections (Steele and Twenhafel, 2010), pulmonary pathology is not typically emphasized in experimental models. In contrast, in our model, RNV-infected mice exhibited overt signs of lung inflammation, including cellular infiltrates consistent with developing pneumonia (**Figure 6**), revealing the value of our model for investigating viral target cells in the lung. The observed septal thickening and inflammatory remodeling may reflect an early tissue response to viral-induced injury, potentially contributing to impaired pulmonary function during severe infection. Notably, in the only confirmed human case of RNV infection to date, bibasilar crackles were reported during clinical examination (Mafayle et al., 2023), which may indicate lower respiratory tract inflammation. These findings suggest that pulmonary involvement could represent an underrecognized aspect of RNV infection, particularly in settings of host susceptibility, although the extent to which this experimental phenotype reflects human disease remains to be determined.

Following early dissemination and peripheral replication, RNV infection in this model progressed toward sustained CNS involvement. Infectious virus in brain homogenates peaked at 5 dpi (**Figure 3**), temporally aligning with astrocyte activation and the onset of neurological symptoms. GFAP upregulation and increased astrocytic density are consistent with patterns described for neurotropic alphaviruses, in which glial activation accompanies peak viral replication and contributes to the inflammatory milieu of the infected CNS (Peng et al., 2013; Phelps et al., 2023). Although we did not perform co-localization or morphometric analyses, the concurrence of high viral titers and pronounced astrogliosis supports a role for glial activation in shaping the observed neuroinflammatory environment. Astrocytes are confirmed to be susceptible to infection by members of the Togaviridae family and are key mediators of the innate immune response via cytokine and chemokine release (Bengue et al., 2021; Geddes et al., 2021; Ronca et al., 2016). While the temporal relationship between this glial response and neurological injury remains to be determined, available evidence suggests that astrocyte-driven inflammation may precede and shape downstream neurodegeneration (Jorgačevski and Potokar, 2023).

This interpretation is further supported by the cytokine profile observed in brain homogenates (**Figure 11**), characterized by elevated IFN- γ, IL-6, and MCP-1/CCL2 during peak CNS infection. Elevated levels of IFN-γ observed in symptomatic mice suggest a central role in orchestrating this response, as it is known to disrupt barrier integrity, recruit immune effector cells, and alter non-immune cell function across tissues, including the CNS (De Benedetti et al., 2021). Similarly, IL-6 has been implicated in sustained neuroinflammation and blood-brain barrier (BBB) dysfunction during viral encephalitis, often acting synergistically with TNF and IFN-γ (Hirano, 2021; Hu et al., 2021; Velazquez-Salinas et al., 2019). MCP-1/CCL2, a key chemokine for leukocyte recruitment, was among the most strongly upregulated inflammatory mediators in our analysis, suggesting a prominent role in shaping the inflammatory environment. This chemokine has been shown to mediate BBB permeability and neuroinflammatory injury in alphavirus infections (De Oliveira Souza et al., 2024). Similar patterns of viral replication, glial activation, and cytokine induction have been reported in the attenuated VEEV TC-83 model, where the inflammatory milieu has been proposed to shape not only pathology but also within-host viral evolution (Williams et al., 2023). Comparable cytokine signatures have been described in models of virulent VEEV infection, where neuropathology occurs not only in regions with detectable viral antigen but also in areas of reactive gliosis, underscoring the paradoxical role of inflammation as both antiviral and pathogenic (Sharma and Knollmann-Ritschel, 2019). Together, the temporal association between viral replication, astrocyte activation, and pro-inflammatory cytokine induction suggests the establishment of an immunopathological loop that may contribute to neuronal injury.

Consistently, neuropathological examination revealed marked neurodegeneration in the cortex and striatum at later stages of infection (**Figure 9**), placing neuronal damage downstream of early glial and cytokine responses. HE-stained sections further demonstrated meningoencephalitis and perivascular cuffing, vacuolization, and tissue disorganization (**Figures 7**, **8**), supporting the olfactory bulb as a major CNS entry route for VEEV after peripheral infection (Cain et al., 2024; Charles et al., 1995; Phillips et al., 2016; Steele and Twenhafel, 2010).

In sum, our study provides the first experimental characterization of RNV pathogenesis in a susceptible mouse model, revealing that this enzootic and historically neglected member of the VEEV complex can elicit significant neuroinflammatory and degenerative outcomes. By integrating diverse parameters within a single experimental framework, this work identifies key pathological checkpoints -such as cytokine elevation and splenic collapse- that warrant further mechanistic investigation. While specific aspects of RNV biology, including the precise routes of neuroinvasion, the cellular targets of infection within affected tissues, or the mechanisms underlying lymphoid architectural disruption, remain to be fully elucidated, this study establishes a necessary foundational baseline. In particular, future studies incorporating tissue-level viral detection and quantitative spatial analysis of immune cell redistribution will be important to further dissect the pathological processes described here. Notably, because this model utilizes immunologically immature mice and human RNV cases remain clinically under-characterized, a direct translational interpretation of these findings requires caution. However, within this broader context, our data challenge long-held assumptions regarding the clinical insignificance of RNV, illustrating how host developmental stages can unmask the pathogenic potential of otherwise neglected viruses.

## Data Availability

The original contributions presented in the study are included in the article/**Supplementary Material**. Further inquiries can be directed to the corresponding author.
